# Pain management and patient education interventions to increase physical activity in people with intermittent claudication (PrEPAID): a feasibility randomised controlled trial in the UK

**DOI:** 10.1136/bmjopen-2025-105563

**Published:** 2025-07-22

**Authors:** Chris Seenan, Ukachukwu Abaraogu, Philippa Margaret Dall, Lesley Gilmour, Garry Tew, Wesley Stuart, Andrew Elders, Julie Brittenden

**Affiliations:** 1 Faculty of Health Sciences and Sport, University of Stirling, Stirling, UK; 2 Institute for Applied Social and Health Research, University of the West of Scotland, Paisley, Scotland, UK; 3 School of Health and Life Sciences, Glasgow Caledonian University, Glasgow, Glasgow, UK; 4 NHS Greater Glasgow and Clyde, Glasgow, Scotland, UK; 5 Institute for Health and Care Improvement, York St John University, York, UK; 6 Glasgow Caledonian University, Glasgow, UK; 7 Institute of Cardiovascular and Medical Sciences, University of Glasgow, Glasgow, Glasgow, UK; 8 Vascular Surgery, NHS Greater Glasgow and Clyde, Glasgow, Glasgow, UK

**Keywords:** Exercise, VASCULAR MEDICINE, Electric Stimulation Therapy, Patient-Centered Care, Behavior, Feasibility Studies

## Abstract

**Objectives:**

To explore the feasibility and acceptability of pain management (transcutaneous electrical nerve stimulation (TENS)) and patient education (PE) to increase physical activity in people with peripheral arterial disease and intermittent claudication (IC).

**Design:**

Feasibility randomised controlled trial with embedded process evaluation.

**Setting:**

One secondary care UK vascular centre.

**Participants:**

56 community-dwelling adults with a history of stable IC and ankle-brachial pressure index ≤0.9 were recruited via claudication clinics.

**Interventions:**

Participants randomised to 6 weeks of: TENS+PE, TENS, Placebo TENS+PE or Placebo TENS. PE was a 3-hour workshop plus three follow-up phone calls. The TENS machine was worn during walking (TENS: 120 Hz, 200 μs, intensity ‘strong but comfortable’; Placebo TENS: intensity below sensation threshold).

**Outcomes:**

Primary feasibility outcomes included rates of recruitment, retention and adherence. Acceptability of the intervention and trial procedures was explored with semistructured interviews. Measures of walking capacity, walking behaviour, quality of life, disease perception and pain were recorded at baseline, end of intervention (6 weeks) and follow-up (3 months).

**Results:**

56 participants were randomised from 95 who completed baseline screening. Of the 39 excluded, 97% (38/39) had >20% variability in absolute claudication distance. All participants received their allocated intervention. Outcome completion was 91% at 6 weeks and 80% at 3 months. Attendance at group education was 96% with 63% taking follow-up phone calls. Compliance with TENS was 70% according to participant-completed logs. Interviewed participants (n=9) were generally positive about the acceptability of the interventions and trial procedures; however, experience of TENS use was mixed. Some participants were dissatisfied with the size of the device and electrode wires.

**Conclusions:**

The PrEPAID (Pain management and Patient Education for Physical Activity in Intermittent claudication) trial was feasible to run; however, 40% of potential participants were excluded at screening due to issues of research fidelity rather than participant suitability or willingness to participate. A future definitive trial should consider a revised primary outcome measure and smaller wireless TENS machines.

**Trial registration number:**

ClinicalTrials.gov, NCT03204825. Registered on 2 July 2017.

**Trial funding:**

Chief Scientist Office, Scottish Government. Translational grant award (TCS/16/55).

**STRENGTHS AND LIMITATIONS OF THIS STUDY**

- A feasibility trial developed following the Medical Research Council Framework for the development of complex interventions.
- Acceptability and feasibility were established using a multi-lens perspective drawing on quantitative, qualitative and trial procedure data.
- Effectiveness of intervention components will need to be established in a full multicentre trial.

## Introduction

 Peripheral arterial disease (PAD) affects more than 3 million people in the UK.^1^ People with PAD have an increased risk of cardiovascular disease mortality^2 3^ and all-cause mortality,^2^ compared with those without the disease.^3^ Approximately 40–75% of people with PAD experience intermittent claudication (IC),^4^ which manifests as pain, fatigue or cramping in the lower limb(s) occurring during activity, such as walking, and relieved by rest.^5^ IC is chronic and progressive, leading to disability,^6^ increasing impairment in physical function^7 8^ and poorer health-related quality of life.^3 9^

Clinical guidelines recommend that people with PAD should receive the same secondary prevention as those with coronary artery disease,^10 11^ including management of blood pressure, cholesterol, smoking cessation and eating a healthy diet and physical activity (PA). The National Institute for Health and Care Excellence recommends supervised exercise therapy (SET) as the primary treatment for IC,^11^ which has been shown to lead to improvements in walking distance of people with IC on a treadmill. However, SET is not routinely available both across the UK and worldwide due to funding, availability of staffing, facilities and expertise.^12^,^16^ Where supervised exercise is available, uptake and adherence are low.^14 17^

Lack of self-efficacy, linked to limited understanding of the disease and uncertainty about the importance of exercise, has been shown to be a major barrier to exercise uptake in this population.^18^ Also, for people with IC to maximise the benefits of improved walking ability and secondary prevention, they are recommended to exercise past the point when they first feel pain, which represents another barrier to engagement.^19^ These barriers of pain and lack of knowledge suggest that pain management and patient education (PE) might be important components of interventions to enhance uptake and adherence to exercise recommendations in individuals with PAD and IC.

Previously, a pilot evaluation (n=14) of a person-centred, education intervention for people with PAD and IC with the aim of educating them about their condition, improving ownership and promoting self-managed walking (Structured EDucation for Rehabilitation in Intermittent Claudication; SEDRIC).^20^ Found that treadmill walking distances and quality of life improved after 6 weeks (30% and 32%, respectively) and there was a trend for patients to increase their daily PA (approximately 8% change from baseline).^20^ Transcutaneous electrical nerve stimulation (TENS), a low-cost, European Conformity-marked non-invasive pain management device, has been shown to significantly increase pain threshold, tolerance and endurance compared with placebo in healthy volunteers^21^ and improve absolute claudication distance (ACD) by 40% when used by people with IC while exercising on a treadmill (n=40) compared with placebo.^22^ Although patient-centred education (SEDRIC) and TENS have both demonstrated potential to improve PA in people with IC, the use of these components in combination has not previously been evaluated. Therefore, this feasibility randomised controlled trial (RCT) aimed to examine the feasibility of conducting a definitive RCT of TENS with and without patient-centred education and the acceptability of these interventions for people with PAD and IC. The trial takes the form of an external pilot study as there is limited uncertainty, but the processes of a definitive trial require evaluation. Also, the feasibility and acceptability of the interventions have not been investigated.^23^

## Methods

### Study objectives

The overall aims of the study were twofold: (1) to explore the feasibility of conducting a definitive RCT of TENS with and without patient-centred education in people with PAD and IC (ie, recruitment and retention rates, intervention uptake and adherence, safety, outcome completion rates and collect outcome information to allow future sample size estimation); and (2) to explore how acceptable (a) the interventions (TENS and patient-centred education) and (b) the trial procedures are to people with PAD and IC.

### Trial Design

**Figure 1:**
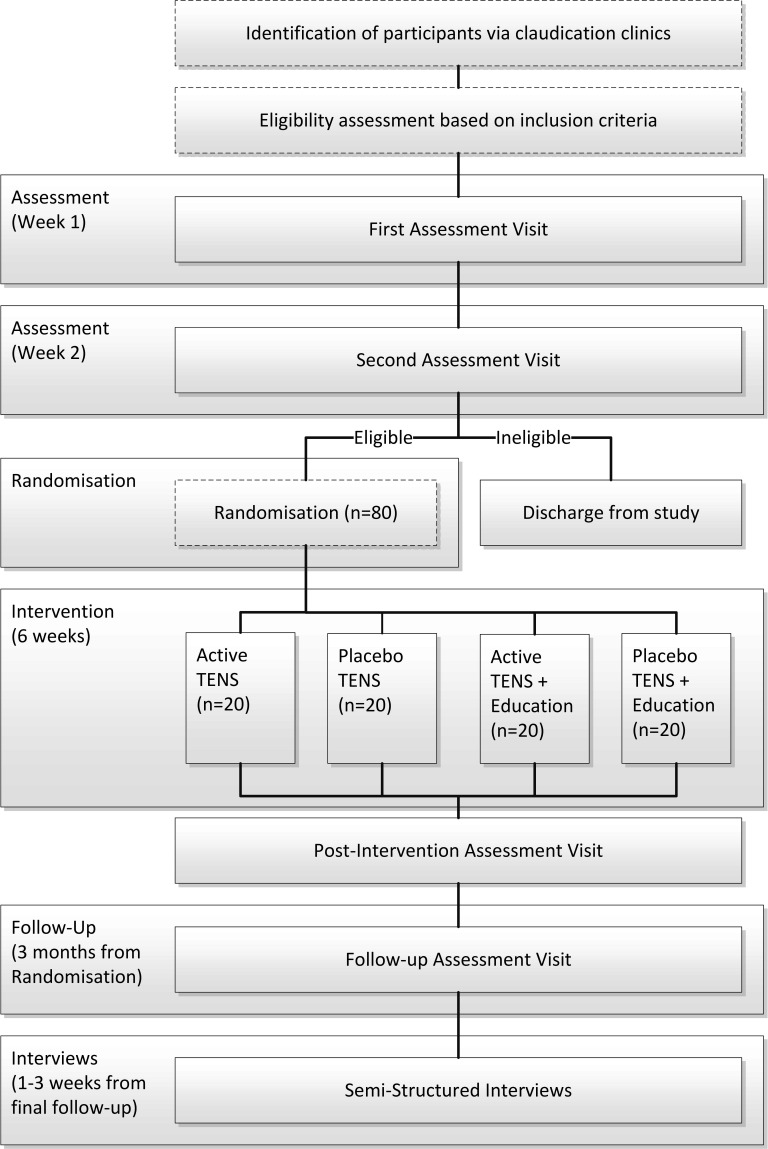
Flow diagram of trial procedures including identification, screening, randomisation, study design, assessments and follow-up. TENS, transcutaneous electrical nerve stimulation.

The PrEPAID trial (Pain management and Patient Education for Physical Activity in Intermittent claudication) was a single-centre, open, feasibility RCT with embedded process evaluation. The methods are detailed in the published protocol,^24^ summarised below and in figure 1.

### Participants

Patients (aged 40–85 years) with a history of stable IC (self-reported walking distance not substantially changed within the past 3 months) and an ankle-brachial pressure index (ABPI) ≤0.9 were eligible to participate. Potentially eligible patients were identified by reviewing claudication clinic lists from one National Health Service (NHS) board in Scotland and either approached in person at their clinic visit or contacted by post. Those who were willing to participate returned an opt-in sheet by post. The research team then contacted them by phone to arrange the first assessment visit where the informed consent process was completed.

### Interventions

#### Transcutaneous electrical nerve stimulation

Participants were provided with a TENS device (MTR+Dolito, EME Services, Manchester, UK) and one-to-one training (approximately 30 mins duration) at the intervention visit. Two self-adhesive carbon rubber electrodes measuring 5×5 cm (StiMus Hydrogel Premium Self-Adhesive Electrodes, EME Services) were attached to the TENS unit via the manufacturer’s leads. Electrode placement was determined by the area of pain reported by the participant during walking, with two electrodes placed at least 2 cm apart either side of the site of pain. Those with bilateral claudication were advised to wear the device either on both limbs or the most painful limb and provided training on using four electrodes. All participants were encouraged to experiment with different electrode placements to find the optimal application.

Participants were asked to wear the device every day for 6 weeks, as often as they could when they were awake, and to switch it on when they were standing/walking or about to engage in activity which they anticipated would trigger their IC pain. They were instructed to repeat this as often as needed during daily activity.

#### Active TENS

Those randomised to receive Active TENS were provided with a device locked to settings of 120 Hz, 200 μs, but free to adjust the intensity to a ‘strong but tolerable’ level.^22^

#### Placebo TENS

Participants allocated to Placebo TENS were provided with the same model of device and instructions for use as those in the Active TENS group, except that the stimulation dose was safely altered to produce non-therapeutic, ineffective stimulation (6 mA). For the purposes of blinding, participants were told that different dosages of TENS were being tested and for some of these dosages they might not feel anything even though the device was working.

### Patient-centred education

The previously developed SEDRIC structured group education was adopted and delivered to those randomised to the education groups. The education intervention consisted of a one-off, 3-hour group workshop (4–6 participants), facilitated by two trained educators and followed by phone calls every 2 weeks to review progress.

The facilitators were qualified healthcare professionals (physiotherapist and nurse), and both had completed the Diabetes Education and Self-Management for Ongoing and Newly Diagnosed core training; associated reading and demonstrated understanding of the SEDRIC curriculum. Workshop delivery was practised and quality assured by experts prior to delivering to the participants.

The structured education aimed to modify participants’ illness beliefs and perceptions about their condition by educating them on disease pathology and management philosophy. During the workshop, each participant was supported to set goals for walking based around daily steps recorded using a pedometer (G-Sensor 2027 3D Pocket Pedometer), and to develop an action plan regarding how these goals would be met. Participants were encouraged to repeat this process for each new walking goal via biweekly phone calls from one of the educators (CS) during which their progress, barriers and challenges were discussed, and new walking goals set. This conversation was guided by the PrEPAID follow-up phone call proforma (see online supplemental file 1).

### Sample size and randomisation

Potential participants attending the vascular outpatient clinics within NHS Greater Glasgow and Clyde were identified. These individuals were either approached face-to-face at their vascular clinic appointment or via post and provided with the study information. Those who wished to find out more about the study were asked to return a prepaid response slip, and a member of the study team contacted them by phone to address any questions and, if appropriate, arrange a time to attend the clinical research facility to complete informed consent and further eligibility screening.

Eligible participants who had completed the baseline assessments were randomly allocated to the trial arms. A central and independent randomisation facility based at the Robertson Centre for Biostatistics (University of Glasgow) allocated the randomised intervention(s) per participant. A simple fixed block design was used to allocate participants to the groups. The participants, outcome assessors and data analyst were blinded to group assignment.

As this is an external pilot study, an a priori sample size calculation was not required. However, we did use a sample size calculation based on previous pilot data from the two arms of the intervention to ensure that our selected sample size was suitable, minimising the burden of conducting the study while collecting sufficient data for future sample size calculations.^25^ At an 80% power and a two-tailed 5% significance level, the sample size calculation indicated that 64 participants (16 per group) were needed to detect an effect size of 1.0 SD for the primary outcome (ACD) in the Active TENS group compared with the Placebo TENS control, based on insights from previous pilot trials.^20 22^ To account for potential attrition, a recruitment target of 80 participants was set.

### Outcomes

To assess feasibility, recruitment rates, reasons for non-eligibility and non-recruitment of eligible patients were recorded along with retention throughout the trial and reasons for withdrawal. Adverse events were monitored, recorded, managed and followed up. Intervention uptake (log of TENS use (participant diary and device record) and engagement with education) was measured, and questionnaires were used to record acceptability of interventions and TENS blinding fidelity. Outcome completion rate for all outcomes (number of days the activPAL is worn, treadmill test completion, patient-reported outcome measures at each outcome time point) was assessed.

As well as the feasibility outcomes that were being assessed in this feasibility study, we collected outcomes we assume would form an assessment of efficacy in a future trial. These were measured at baseline, at the end of the 6-week intervention and at 3 months follow-up. ACD (metres) (assumed primary outcome of future trial) was measured using the Gardner treadmill protocol.^6^ Other outcomes included: initial claudication distance (ICD; metres);^6^ daily PA via activPAL activity monitor (total number of: steps; upright events; walking events, and the event-based claudication index (EBCI));^26^ disease-specific quality of life (Intermittent Claudication Questionnaire (ICQ)),^27^ generic quality of life (General Quality of Life Questionnaire (Short Form); SF-36);^28^ pain quality (McGill Pain Questionnaire; MPQ)^29^ average pain intensity (Visual Analogue Scale);^30^ illness beliefs and psychosocial determinants of health and behaviour (Brief Illness Perception Questionnaire,^31^ Geriatric Depression Scale: Short Form (GDS-SF)^32^ and Pain Self-Efficacy Questionnaire (PSEQ)^33^).

### Impact of the COVID-19 pandemic

All quantitative data collection and delivery of interventions were completed prior to the UK national lockdowns in March 2020. However, it was not possible to arrange the planned focus groups to explore participant experiences of the interventions and study procedures. Individual, semistructured interviews were conducted via phone as an amendment to the protocol.

### Qualitative interviews

Participants were invited to take part in one interview after the final follow-up visit to contribute to the process evaluation for the trial. All participants were approached, and participants from different groups were purposively selected. Semistructured telephone interviews were conducted to explore the acceptability of the interventions and study procedures (Topic Guide available in online supplemental file 2). All interviews were digitally recorded with permission, transcribed verbatim and coded using NVivo (V.20) and analysed using framework analysis using themes from the theoretical framework of acceptability.^34^

### Statistical methods

The Robertson Centre for Biostatistics, part of the Glasgow Clinical Trials Unit, managed the trial data. Statistical analysis was led by the study statistician (AE) at Glasgow Caledonian University; the statistician was blinded to group allocation.

Baseline variables are summarised using descriptive statistics. The feasibility, acceptability, adverse events data and protocol and intervention adherence data are summarised by randomised group and overall using descriptive statistics. Participant outcomes are summarised descriptively by group and time point, including the extent of missing data. The trial protocol reported planned statistical analysis including exploring between-group and within-group differences; however, ultimately, no formal hypothesis tests were conducted due to the focus being on feasibility.

All outcomes are summarised descriptively at each time point, with the SD being estimated alongside a CI in line with current guidance on estimation of SD from feasibility trials^35^ and Cohen’s d effect size. Intention-to-treat analysis was performed for all participants. Due to the limited sample size, multiple imputations were not used, and missing values were adjusted to the last observation carried forward.

### Patient and public involvement

Two patient and public involvement representatives provided input as members of the trial management group. They contributed to the development of study documentation and provided suggestions on enhancements to the protocol. During analysis, they acted as additional researchers, checking and providing comments on the analysis of the participant interviews.

## Results

### Participant flow/recruitment

**Figure 2:**
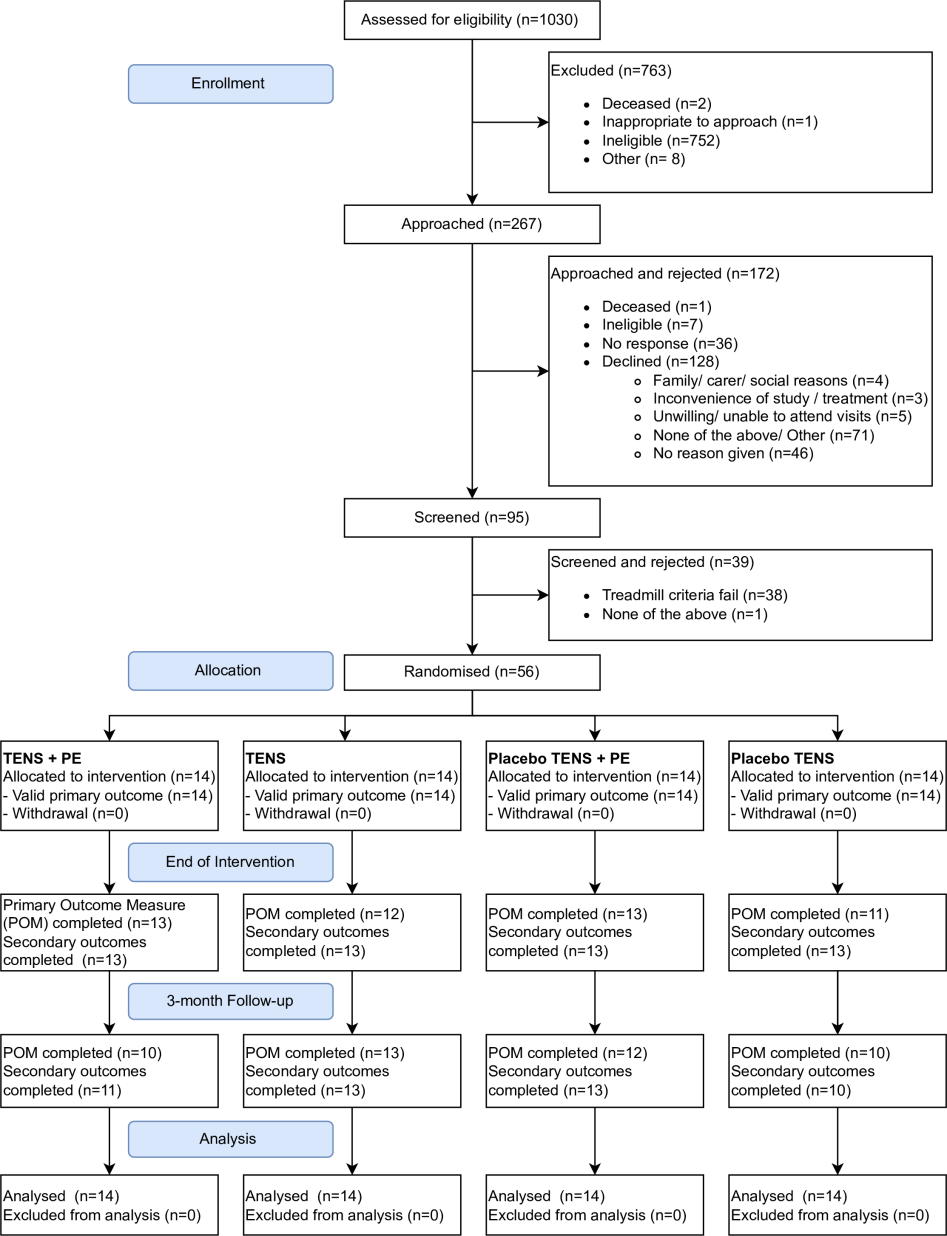
CONSORT diagram. CONSORT, Consolidated Standards of Reporting Trials; PE, patient education; TENS, transcutaneous electrical nerve stimulation.

A CONSORT (Consolidated Standards of Reporting Trials) diagram shows the flow of participants through the trial (figure 2). The trial was open for recruitment between August 2017 and March 2020. In total, 1030 people were screened for eligibility; 763 (74%) were ineligible, mainly because they were on the clinic lists due to other conditions, for example, abdominal aortic aneurysm or venous disease (n=343, 45% of ineligible total) or had critical limb ischaemia (n=122, 16%). There were 172 individuals who either did not consent to participate or did not respond to the invitation. Common reasons for declining are noted in figure 2.

39 participants did not pass screening, 38 (97%) due to having a greater than 20% variance in ACD between the first and second screening visits (1 week apart). The other participant who did not pass screening was unable to walk on the treadmill.

### Participants

Table 1 shows the baseline characteristics of all participants. Mean age of those who were randomised was 66 years (SD 8.6) and 75% (n=42) were male. Mean time since diagnosis of PAD was 2.8 years (SD 4.3), 59% (n=33) had femoral disease and the mean ABPI was 0.69 (SD 0.20). Generally, group characteristics were well balanced across groups, although participants in the TENS+PE group had lived with PAD and IC for a longer time (5.2 vs between 1.4 and 2.8 years) and had more proximal disease (93% femoral and iliac vs 71%) than those in the other groups. There were some other differences in lifestyle behaviours (eg, smoking; 50% of participants in the TENS+PE group were current smokers compared with 7% in the Placebo TENS+PE group) and medical history, potentially due to small group sizes.

**Table 1:** Baseline characteristics for all participants by group and overall

	TENS	Placebo TENS	Placebo TENS+PE	TENS+PE	All randomised	Not randomised
Core demographic and clinical characteristics						
Age at recruitment, mean (SD), N	65.1 (9.7), 14	69.1 (10.1), 14	64.0 (5.5), 14	66.9 (8.3), 14	66.3 (8.6), 56	66.7 (8.5), 39
Sex (% male), per cent, n/N	71, 10/14	93, 13/14	71, 10/14	64, 9/14	75, 42/56	69, 27/39
Body mass index, mean (SD), N	26.4 (4.7), 14	27.9 (3.9), 14	30.2 (5.5), 14	29.4 (4.3), 14	28.5 (4.8), 56	27.5 (5.4), 39
Ankle-brachial pressure index, mean (SD), N	0.70 (0.21), 14	0.74 (0.28), 14	0.67 (0.15), 14	0.66 (0.14), 14	0.69 (0.20), 56	0.61 (0.14), 39
Peripheral artery disease						
Time since diagnosis (years), mean (SD), N	1.4 (1.4), 14	2.8 (2.6), 14	1.9 (1.7), 14	5.2 (7.6), 14	2.8 (4.3), 56	3.6 (5.4), 39
Time since diagnosis (categories), per cent, n/N						
<3 months	7, 1/14	0, 0/14	0, 0/14	0, 0/14	2, 1/56	3, 1/39
≥3 to <6 months	14, 2/14	7, 1/14	21, 3/14	14, 2/14	14, 8/56	23, 9/39
≥6 to <12 months	36, 5/14	21, 3/14	29, 4/14	14, 2/14	25, 14/56	21, 8/39
≥12 months	43, 6/14	71, 10/14	50, 7/14	71, 10/14	59, 33/56	54, 21/39
Disease type, per cent, n/N						
Iliac	21, 3/14	0, 0/14	14, 2/14	36, 5/14	18, 10/56	13, 5/39
Femoral	50, 7/14	71, 10/14	57, 8/14	57, 8/14	59, 33/56	59, 23/39
Popliteal	14, 2/14	7, 1/14	14, 2/14	7, 1/14	11, 6/56	15, 6/39
Other	14, 2/14	21, 3/14	14, 2/14	0, 0/14	13, 7/56	13, 5/39
Intermittent claudication						
Time since diagnosis (years), mean (SD), N	1.4 (1.4), 14	2.1 (1.6), 14	1.9 (1.7), 14	5.2 (7.6), 14	2.6 (4.2), 56	3.5 (5.4), 39
Time since diagnosis (categories), per cent, n/N						
<3 months	7, 1/14	7, 1/14	0, 0/14	0, 0/14	4, 2/56	0, 0/39
≥3 to <6 months	14, 2/14	0, 0/14	21, 3/14	14, 2/14	13, 7/56	28, 11/39
≥6 to <12 months	36, 5/14	29, 4/14	29, 4/14	14, 2/14	27, 15/56	21, 8/39
≥12 months	43, 6/14	64, 9/14	50, 7/14	71, 10/14	57, 32/56	51, 20/39
Limb affected, per cent, n/N						
Left	21, 3/14	36, 5/14	36, 5/14	29, 4/14	30, 17/56	13, 5/39
Right	36, 5/14	14, 2/14	29, 4/14	21, 3/14	25, 14/56	15, 6/39
Both	43, 6/14	50, 7/14	36, 5/14	50, 7/14	45, 25/56	72, 28/39
Fontaine classification, per cent, n/N						
I	7, 1/14	0, 0/14	0, 0/14	0, 0/14	2, 1/56	0, 0/39
II	93, 13/14	100, 14/14	100, 14/14	100, 14/14	98, 55/56	100, 39/39
III	0, 0/14	0, 0/14	0, 0/14	0, 0/14	0, 0/56	0, 0/39
IV	0, 0/14	0, 0/14	0, 0/14	0, 0/14	0, 0/56	0, 0/39
Cardiac risk factors per cent (n/N)						
Diabetes mellitus,	29, 4/14	21, 3/14	14, 2/14	7, 1/14	18, 10/56	38, 15/39
Family history of IHC/CVA/BP	64, 9/14	36, 5/14	50, 7/14	36, 5/14	46, 26/56	59, 23/39
Hypercholesterolaemia	21, 3/14	14, 2/14	43, 6/14	36, 5/14	29, 16/56	31, 12/39
Hypertension	50, 7/14	57, 8/14	43, 6/14	64, 9/14	54, 30/56	51, 20/39
Alcohol excess, per cent (n/N)						
Current	7, 1/14	14, 2/14	29, 4/14	14, 2/14	16, 9/56	13, 5/39
Previous	21, 3/14	14, 2/14	7, 1/14	7, 1/14	13, 7/56	18, 7/39
Never	71, 10/14	71, 10/14	64, 9/14	79, 11/14	71, 40/56	69, 27/39
Smoking, per cent (n/N)						
Current	29, 4/14	29, 4/14	7, 1/14	50, 7/14	29, 16/56	28, 11/39
Previous	64, 9/14	43, 6/14	86, 12/14	43, 6/14	59, 33/56	59, 23/39
Non-smoker	7, 1/14	29, 4/14	7, 1/14	7, 1/14	13, 7/56	13, 5/39
Medical history, per cent (n/N)						
Ischaemic heart disease	21, 3/14	7, 1/14	21, 3/14	7, 1/14	14, 8/56	15, 6/39
Myocardial infarction	14, 2/14	14, 2/14	14, 2/14	0, 0/14	11, 6/56	21, 8/39
Left ventricular hypertrophy	0, 0/14	0, 0/14	0, 0/14	0, 0/14	0, 0/56	3, 1/39
Present drug history, per cent (n/N)						
Statins	93, 13/14	86, 12/14	86, 12/14	93, 13/14	89, 50/56	79, 31/39
Antiplatelets	57, 8/14	86, 12/14	86, 12/14	64, 9/14	73, 41/56	62, 24/39
Anticoagulants	14, 2/14	14, 2/14	14, 2/14	14, 2/14	14, 8/56	5, 2/39
Walking distances, mean (SD), N						
Initial claudication distance (ICD) (metres)	84.4 (78.2), 14	126.1 (165.2), 14	130.2 (129.6), 14	111.7 (162.3), 14	113.1 (135.7), 56	71.2 (72.8), 38
Absolute claudication distance (ACD) (metres)	243.7 (240.5), 14	251.3 (266.5), 14	370.9 (285.3), 14	318.4 (321.6), 14	296.1 (277.3), 56	207.6 (179.9), 38
Physical activity measures, mean (SD), N						
Average daily number of steps (’000 s)	7.5 (7.1), 14	4.9 (2.1), 14	6.0 (2.8), 14	5.1 (3.9), 14	5.9 (4.4), 52	5.2 (2.6), 36
Average daily number of upright events	7.0 (2.9), 14	6.1 (2.7), 14	6.6 (2.9), 14	6.1 (1.8), 14	6.4 (2.5), 52	8.2 (3.4), 36
Average daily number of walking events	50.0 (24.0), 14	50.2 (21.2), 14	53.6 (26.0), 14	46.4 (19.1), 14	50.0 (22.2), 52	50.4 (20.1), 36
Event-based claudication index	7.3 (2.8), 14	8.9 (4.4), 14	8.4 (4.3), 14	8.5 (6.3), 14	8.3 (4.6), 52	6.8 (2.7), 36
Participant-reported measures, mean (SD), N						
SF-36 Physical Component Score	34.4 (7.5), 14	32.5 (8.9), 14	37.3 (5.2), 14	38.1 (6.0), 14	35.6 (7.2), 56	33.0 (5.5), 39
SF-36 Mental Component Score	23.9 (11.3), 14	22.7 (11.7), 14	25.3 (12.7), 14	24.6 (13.9), 14	24.1 (12.2), 56	27.0 (10.4), 39
Intermittent Claudication Questionnaire	53.1 (16.0), 14	53.2 (15.7), 14	52.2 (15.4), 14	47.6 (16.0), 14	51.5 (15.5), 56	45.8 (14.9), 39
Geriatric Depression Scale (Short-Form)	6.6 (4.2), 14	5.1 (3.9), 14	7.1 (4.6), 14	6.4 (4.7), 14	6.3 (4.3), 56	7.5 (4.7), 39
McGill Pain Questionnaire	30.6 (13.6), 14	22.2 (13.1), 14	31.2 (14.4), 14	29.4 (10.6), 14	28.4 (13.1), 56	31.1 (12.0), 37
Pain Self-Efficacy Questionnaire	35.8 (13.7), 14	42.4 (14.3), 14	36.5 (14.8), 14	35.2 (13.9), 14	37.5 (14.1), 56	31.2 (16.0), 39
Pain intensity (Visual Analogue Scale)	6.3 (2.3), 14	6.2 (2.8), 14	6.6 (2.4), 14	5.5 (2.6), 14	6.2 (2.5), 56	6.7 (2.7), 39
Brief Illness Perception Questionnaire	47.2 (11.9), 14	43.4 (10.1), 14	49.8 (9.7), 14	49.3 (10.4), 14	47.4 (10.6), 56	52.6 (11.6), 39

BP, blood pressure; CVA, cerebrovascular accident; IHC, inheritied heart conditions; PE, patient education; SF-36, Short Form-36; TENS, transcutaneous electrical nerve stimulation.

Mean ACD was 296 m (SD 277.3) in all randomised participants at baseline. Mean ICD was 113 m (SD 135.7), mean number of steps per day was 5900 (SD 4400) and mean ICQ score was 52 (SD 15.5). In general, group measures at baseline were similar, although the TENS only group had a shorter ICD (84 m vs 111–130 in the other groups) and greater average steps and upright events per day (7500 vs 4900–6000 and 7.0 vs 6.1–6.6, respectively). Otherwise, the TENS+PE group had a lower score on the ICQ (48 vs 52–53) and the Placebo TENS group had lower scores on the GDS-SF, MPQ and PSEQ (see table 1).

### Delivery of interventions, adherence rates and adverse events

All participants received the intervention they were allocated to. Compliance with TENS (defined in the protocol as: ≥30 min/day, ≥3 days/week, ≥3 weeks) was 70% according to participant-completed logs. Usage of TENS was also objectively recorded using the internal memory of the device which measured compliance as 28%, but with substantial missing data due to device errors. Attendance at the group education session for those allocated to education intervention groups was 96% and 63% completed the follow-up phone calls.

One serious adverse event was recorded during the trial. Abnormal and altered ECG findings were observed during treadmill assessment in one participant prior to randomisation. Testing was halted, and this participant was therefore excluded from further participation in the trial. Three minor adverse events were reported throughout the study, all relating to a slight itching reaction to the TENS electrodes.

### Numbers analysed

10 participants withdrew from the trial overall. One did not attend the second screening visit and could not be contacted. One participant was unable to continue as they were listed for endovascular surgery. One participant fractured their ankle and was unable to continue in the study, and one other moved abroad during the study and thus had to withdraw. The other six participants either did not provide a reason for withdrawing or did not respond to efforts to contact them. The participants that withdrew came from all four groups (TENS+PE=3; TENS=1; Placebo TENS+PE=2; Placebo TENS=3).

Primary outcome measure completion for those who remained in the study was 100% (49/49) for end of intervention and 98% (45/46) for 3-month follow-up (one participant was unable to complete treadmill measures but completed all other outcomes). Primary outcome completion for all randomised was therefore 87.5% (49/56) at end of intervention and 80% (45/56) at 3-month follow-up.

### Outcomes and estimation

Data from the primary outcome are presented in tables2  3. There was substantial variation at each time point within each group as demonstrated by the SD and wide 95% CI. The average distance walked was greater in the Placebo TENS and education group at baseline, end of intervention and follow-up. Data for all outcomes at the end of intervention and 3-month follow-up are available in online supplemental additional file 1.

**Table 2:** Absolute claudication distance presented by intervention group (intention-to-treat)

ACD (metres)	TENS+PE	TENS	Placebo TENS+PE	Placebo TENS
Baseline				
n (%)	14 (100)	14 (100)	14 (100)	14 (100)
Mean	318.4	243.7	370.9	251.3
SD (95% CI)	321.6 (132.7 to 504.1)	240.5 (104.8 to 382.6)	285.3 (206.2 to 535.6)	266.5 (97.4 to 405.2)
Median (IQR)	158.3 (369)	182.8 (197)	287.9 (368)	138.1 (295)
Minimum, maximum	49, 966	21, 797	45, 966	38, 966
End of intervention				
n (%)	14 (100)	14 (100)	14 (100)	14 (100)
Mean	317.6	229.3	441.7	246.1
SD (95% CI)	284.1 (153.6 to 481.6)	210.1 (107.9 to 350.6)	333.5 (249.1 to 634.3)	259.8 (96.1 to 396.0)
Median (IQR)	187.3 (328)	204.7 (160)	358.1 (528)	139.0 (310)
Minimum, maximum	55, 966	7, 688	28, 966	38, 966
Cohen’s d_s_	**0.00**	**−0.06**	**0.23**	**−0.02**
Follow-up				
n (%)	14 (100)	14 (100)	14 (100)	14 (100)
Mean	316.9	266.9	456.2	238.0
SD (95% CI)	292.4 (148.0 to 485.7)	241.8 (127.3 to 406.5)	324.6 (268.7 to 643.6)	262.5 (86.5 to 389.6)
Median (IQR)	214.1 (384)	225.3 (305)	422.9 (546)	125.6 (268)
Minimum, maximum	55, 966	44, 772	52, 966	33, 966
Cohen’s d_s_	**0.00**	**0.10**	**0.28**	**−0.05**

Cohen’s d_s_ represents change from baseline.

ACD, absolute claudication distance; PE, patient education; TENS, transcutaneous electrical nerve stimulation.

**Table 3:** Absolute claudication distance presented by intervention group (per protocol)

ACD (metres)	TENS+PE	TENS	Placebo TENS+PE	Placebo TENS
Baseline				
n (%)	14 (100)	14 (100)	14 (100)	14 (100)
Mean	326.3	264.1	337.7	301.8
SD (95% CI)	352.5 (74.1 to 578.4)	253.1 (103.3 to 425.0)	272.6 (164.6 to 510.9)	302 (85.7 to 517.8)
Median (IQR)	158.3 (454)	186.9 (237)	270.5 (311)	176.6 (403)
Minimum, maximum	49, 966	21, 797	45, 966	38, 966
End of intervention				
n (%)	13 (93)	12 (86)	13 (93)	11 (79)
Mean	312.1	247.3	410.0	295.4
SD (95% CI)	302.5 (95.6 to 528.5)	220.5 (107.2 to 387.4)	321.8 (205.6 to 614.5)	294.4 (84.8 to 506.0)
Median (IQR)	170.3 (324)	218.6 (161)	358.1 (447)	160.0 (411)
Minimum, maximum	68, 966	7, 688	28, 966	38, 966
Cohen’s d_s_	**−0.04**	**−0.07**	**0.24**	**−0.02**
Follow-up				
n (%)	10 (71)	13 (93)	12 (86)	10 (71)
Mean	311.1	283.5	426.9	284.1
SD (95% CI)	313.7 (86.7 to 535.6)	253.5 (122.5 to 444.5)	312.6 (228.3 to 625.6)	299.9 (69.6 to 498.7)
Median (IQR)	190.9 (403)	225.3 (452)	422.9 (465)	133.7 (412)
Minimum, maximum	60, 966	58, 772	52, 966	33, 966
Cohen’s d_s_	**−0.02**	**0.16**	**0.18**	**0.11**

Cohen’s d_s_ represents change from baseline.

ACD, absolute claudication distance; PE, patient education; TENS, transcutaneous electrical nerve stimulation.

### Process evaluation

18 participants volunteered to participate in the semistructured interviews, nine interviews were completed (20% of final sample). Participants represented all arms of the trial (TENS=1; TENS+PE=2; Placebo TENS+PE=3; Placebo TENS=3). The domains of the theoretical framework of acceptability were used as a priori themes for the framework analysis of the transcripts from the interviews (see table 4 for an overview).

**Table 4:** Overview of acceptability of patient education and TENS as interventions according to the domains of the theoretical framework of acceptability

Theoretical framework of acceptability domain *Description of domain*	Patient education component	TENS component
Affective attitude *How an individual feels about the intervention*	Enjoyable experience. Good to have answers to questions. Enjoyed learning from others.	Mixed experience. Some initially apprehensive. Some awareness of positive experiences of friends/family.
Burden *The perceived amount of effort that is required to participate in the intervention*	Potential to streamline delivery to be more local for some.	Cumbersome and annoying for some, wires and size of unit.
Ethicality *The extent to which the intervention has good fit with an individual’s value system*	Recognised need to be active in the management of their condition. Felt both interventions should be offered on NHS.	
Intervention coherence *The extent to which the participant understands the intervention and how it works*	Most participants understood the need to engage for the intervention to be successful. Some participants understood the benefit of the pedometer to help with goal setting and feedback. Others did not understand the purpose of education.	Most participants understood the need to engage for the intervention to be successful.
Opportunity costs *The extent to which benefits, profits or values must be given up to engage in the intervention*	Time and effort required to participate.	Size of unit affected choice of clothing and sometimes choice of activity. Time and effort required to participate.
Perceived effectiveness *The extent to which the intervention is perceived as likely to achieve its purpose*	Some participants perceived a positive effect. Interventions viewed as more acceptable if perceived to work.	
Self-efficacy *The participant’s confidence that they can perform the behaviour(s) required to participate in the intervention*	One participant felt group discussion might be difficult for some (although not for themselves).	One participant found it difficult to use the device properly and asked for clearer instructions.

The domains and domain descriptions are from the theoretical framework of acceptability.^34^

NHS, National Health Service; TENS, transcutaneous electrical nerve stimulation.

*Affective attitude:* Experience of using TENS was mixed among the participants. Some were fearful or apprehensive prior to using it, but those who were aware of positive experiences from friends or family were not so much, and this commonly related to positive experiences overall. Participants really enjoyed the education intervention and how it was delivered. They felt good to have answers to their questions, an opportunity to discuss their situation and information about what they can do to help their situation. This was perceived as a counterpoint to other healthcare for PAD, where they feel they do not get told anything about their condition, and they felt empowered finding out they could do something about their condition. The aspects they enjoyed were listening to others who are in the same situation and learning/discussing what they can do/planning for this action. Both interventions were associated with a positive affective attitude as the participants felt they helped mentally and physically despite being apprehensive prior to the education session and finding TENS annoying as it was hard to put on.

*Burden:* TENS was found cumbersome and annoying by some participants. Specifically, the wiring and the size of the unit, and some would have preferred a unit that was easier to use, smaller so it could be hidden and wireless. Others found it to be fine in its current form and felt that the slight burden of using the machine was worth it for the pain relief. Some participants felt the education component could be streamlined and maybe delivered more locally, for example, in health centres rather than in the hospital. Others felt that the length was fine as it was important to have space to talk.

*Ethicality:* The overall interpretation from those who were interviewed was that the interventions require some patient ‘engagement’. Participants recognised that they need to be active in the management of their condition, and to ‘lie back and accept it’ would mean no change in function or outcome. Generally, they were willing to engage if it led to improvement. The participants felt that both interventions should be offered on the NHS for people with IC but perhaps via general practice surgeries or more locally.

*Intervention coherence:* Participants mostly seemed to understand that for the interventions to be successful, they need to engage with them actively and have an open mindset. They felt that the pedometers were important because they understood the benefits of having an objective measure of how far they were walking. Some also appeared to understand how the interventions were intended to work, that is, through goal setting and feedback. They mentioned that they understood that the improvements in steps on the pedometers were useful to reinforce the psychological improvement. Others, however, did not seem to understand the purpose of the education. For example, one person expected to find out whether they could get surgery or not.

*Opportunity costs:* Participants highlighted how the size of the TENS unit and the fact they could not hide it affected clothing choice, with some reporting that they either could not wear the clothes they wanted as the device would be visible or not wear the device when wearing certain outfits, for example, a summer dress. They also recognised the time and effort required to participate in the interventions as potentially something that others would struggle with.

*Perceived effectiveness:* Both interventions were considered more acceptable if they had a perceived positive effect. There was a mixed experience of benefit from TENS, with some participants reporting large improvements in walking ability and others saying it did not help them walk any further. Interestingly, however, even if it did not help them walk further, many participants thought it might just be something unique to them and that TENS should still be offered to other people with their condition. Both interventions were associated with physical and psychological improvements, with participants reporting ‘feeling better’ and increasing walking distance. Improvements in other functional activities related to work and socialising because of this improvement in walking capacity were also related back to the interventions positively.

*Self-efficacy:* One participant found it difficult to set the TENS device properly and thus did not use the intervention. This participant felt that the instructions could be clearer. Another participant felt that the prospect of participating in a group discussion might be difficult for some people. They were happy/able to do it themselves, but they thought maybe others would be more nervous.

## Discussion

The aim of this study was to determine the feasibility of an RCT examining the effects of TENS and PE in people with PAD and IC on measures of PA. The key finding is that recruitment and retention rates show it would be feasible to undertake a full-scale trial. This study illustrates the acceptability of TENS and the SEDRIC education intervention for people with PAD, with some suggestions for enhancement, and an initial indication of positive effects reported by participants.

Of the 95 participants who agreed to attend for screening, 41% (n=39) were not randomised and 97% of these (n=38) were due to having a greater than 20% variation in ACD between the baseline assessment visits. This eligibility criterion was implemented within the study to try and recruit participants who walk a consistent distance on the treadmill. We felt this was important as the primary efficacy outcome was ACD, and thus consistency in this measure would allow more accurate evaluation of the predicted small effect of the interventions. Therefore, this eligibility criteria was related to the choice of primary outcome for the trial and not related to the suitability of participants to take part in the trial.

The use of this criteria for eligibility seems not to have achieved its purpose and unfortunately excluded participants who were willing to participate, compromising the external validity of the study. Despite recruiting participants who walked a more reliable distance on the treadmill from 1 week to the next, there was substantial variability in ACD across the sample, illustrated by a relative SD of 94% (table 1). Also, there was considerable variability in the response to interventions indicated by the small effect sizes (tables2  3) with wide 95% CIs in mean change scores (online supplemental additional file 1, tables3^4^). The characteristics of those who did not meet the inclusion criteria are not dissimilar to those who were randomised (table 1). There was a trend in those not randomised to include more individuals who have been living with the condition for longer, experience bilateral claudication, have diabetes and a lower EBCI score (less fragmented walking behaviour), but overall, ACD was similar to those who were randomised (207.6 m vs 296.1 m, respectively). A future RCT should consider revising or removing this eligibility criteria as it has not achieved its aim of reducing variability in the primary outcome and has led to the exclusion of participants who otherwise would have been eligible to participate in the study.

A future RCT may also reconsider the selection of a measure of walking capacity as the primary outcome when exploring the effects of complex intervention(s). Walking capacity (eg, measured via ACD or 6-minute walk test) is often used as the primary outcome in trials with people with PAD as it is suggested to be the most meaningful and relevant outcome for daily function and quality of life.^36^ The interventions studied in this trial can be considered complex interventions as they include multiple behaviour change techniques.^37^ Complex interventions are considered to have proximal and distal indicators of behaviour change, conceptualised within their process evaluation or programme theory.^38 39^ With the interventions tested in this trial, for example, changes in illness beliefs or daily PA behaviours would be considered as proximal indicators. Changes in walking capacity or ABPI would be distal outcomes as they are the cumulative, or additive impact of proximal behaviours.^40^ This relates to the multiple factors that influence walking capacity in people with PAD,^41^ and changes in distal outcomes may only be observed after prolonged engagement with interventions.^42^ In the current study, scores in illness beliefs, quality of life (SF-36 and ICQ), pain self-efficacy and pain quality (MPQ) improve from baseline to end of intervention and 3-month follow-up for those who received the education intervention (online supplemental additional file 1, tables3  4). These data suggest a review of primary outcome selection might be warranted for a future RCT and a potential shift to a ‘proximal indicator’ of the outcome of the interventions. This would align with recent research examining the effects of behavioural interventions in PAD that have used the Illness Beliefs Questionnaire^43^ or steps per day^44^ as primary outcome in their RCTs. If this takes place, future sample size calculation for a definitive trial would need to be based on data from the primary outcome selected.

### Limitations

This study successfully evaluated the feasibility and acceptability of TENS and PE interventions in individuals with PAD and IC; however, several limitations related to the process evaluation should be acknowledged. The process evaluation did not comprehensively explore participants’ experiences with recruitment and consent procedures. Although retention rates were high, the exclusion of 40% of screened individuals due to the ACD variability criterion suggests that future studies should further examine how recruitment strategies and eligibility criteria impact participation. Semistructured interviews did not specifically include questions on the recruitment process, meaning valuable insights into participants’ decision-making, motivations and perceived barriers to participation may have been overlooked. There was also a notable discrepancy between self-reported TENS adherence (70%) and device-recorded adherence (28%), with missing data due to device errors. This raises concerns about recall bias and the reliability of adherence measures. The lack of detailed participant feedback on their experiences using the TENS device and completing adherence logs represents a gap in the process evaluation. A future trial should incorporate more robust adherence tracking methods, such as real-time usage monitoring and in-depth qualitative feedback. While individual interviews provided valuable insights, the absence of planned focus groups due to COVID-19 restrictions limited the depth of participant discussion. Group discussions may have facilitated a richer understanding of shared experiences, peer support mechanisms and perceived intervention effectiveness. Future trials should incorporate multiple qualitative data collection points to capture changes in participants’ perceptions over time. Although TENS adherence was tracked, reasons for non-adherence were not explicitly explored beyond general dissatisfaction with the device’s size and wiring. A deeper exploration of contextual barriers, such as time constraints, discomfort or competing health priorities, could inform future refinements to enhance intervention engagement. Despite efforts to exclude participants with highly variable ACD, substantial intraindividual variability remained. This is a key finding which should inform future trial design. A more behaviourally sensitive primary outcome measure may be appropriate, such as daily step count or illness beliefs, which may be more responsive to the intervention and better aligned with behaviour change mechanisms. There was substantial baseline imbalance in some outcomes, for example, smoking status, which may have biased the results. Future studies could consider stratified or dynamic randomisation techniques to improve baseline comparability. These limitations underscore key areas for refinement in future trials. Enhancing recruitment process evaluation, adherence tracking and qualitative follow-up will strengthen the validity and generalisability of findings.

### Adaptations for a full trial

The key learning from the interviews with participants is that further refinements could enhance the acceptability of both interventions. Participant experience of burden, self-efficacy and generally how they felt about TENS could be improved by using smaller, wireless devices and implementing more extensive training and follow-up. A future trial could adjust the type of TENS devices used and include a more developed protocol for training participants to use the device along with regular follow-up via phone to enhance the experience of using the intervention. Enhancements to the delivery of the education intervention might include delivering the session locally to the participants to make it more accessible and to add additional ‘catch-up’ sessions so that participants can reconnect with the others in their session and further benefit from the social support generated and sharing of progress with their walking goals.

## Conclusions

Our findings provide evidence that a future large-scale RCT is feasible. Most study processes proved acceptable to participants, and we have identified procedures and assessments that may be refined for a future trial. We anticipate these will facilitate participant randomisation and minimise participant burden. The findings of a large-scale RCT would provide important evidence to impact interventions provided to people with PAD and IC and to inform national and international clinical guidelines for management of the condition.

## Supplementary material

10.1136/bmjopen-2025-105563 online supplemental file 1

10.1136/bmjopen-2025-105563 online supplemental file 2

10.1136/bmjopen-2025-105563 online supplemental file 3

## Data Availability

Data are available upon reasonable request.
