# Supplementary material for: Pain management and patient education interventions to increase physical activity in people with intermittent claudication (PrEPAID): a feasibility randomised controlled trial in the UK
Source: BMJ Open. 2025 Jul 22;15(7):e105563. doi: 10.1136/bmjopen-2025-105563 (PMC12306470; doi:10.1136/bmjopen-2025-105563)
Supplement: online supplemental file 1 [file bmjopen-15-7-s001.docx]

Additional File 1: Additional Tables

Additional Table 1: All outcome measures at end of intervention (6 weeks). All presented as Mean (SD), N.

|  | **TENS + PE** | **TENS** | **Placebo TENS + PE** | **Placebo TENS** |
| --- | --- | --- | --- | --- |
| **Walking distances** | | |  |  |
| Initial Claudication Distance (ICD) (metres) | 159.4 (246.2), 13 | 128.0 (103.0), 12 | 243.0 (253.7), 13 | 166.3 (235.8), 11 |
| Absolute Claudication Distance (ACD) (metres) | 288.9 (273.8), 13 | 247.3 (220.5), 12 | 452.7 (344.5), 13 | 286.8 (280.8), 11 |
| **Physical activity measures** |  |  |  |  |
| Mean daily number of steps | 5291 (3909), 10 | 6435 (4429), 12 | 6647 (3866), 12 | 4475 (2651), 10 |
| Mean daily number of upright events | 37.3 (9.7), 10 | 44.1 (21.8), 12 | 38.3 (11.6), 12 | 32.5 (10.9), 10 |
| Mean daily number of walking events | 295.5 (190.3), 10 | 298.7 (167.1), 12 | 287.2 (130.3), 12 | 278.2 (137.4), 10 |
| Mean Event-Based Claudication Index | 8.9 (7.6), 10 | 7.1 (2.9), 11 | 8.7 (5.8), 11 | 8.6 (3.5), 8 |
| **Participant-reported measures** | | | |  |
| SF-36 Physical Component Score | 33.6 (7.7), 13 | 35.3 (7.8), 13 | 32.5 (8.4), 13 | 33.6 (8.0), 13 |
| SF-36 Mental Component Score | 22.5 (10.4), 13 | 20.6 (11.1), 13 | 22.2 (11.7), 13 | 22.2 (11.4), 13 |
| Intermittent Claudication Questionnaire | 57.4 (12.4), 13 | 57.4 (14.4), 13 | 59.5 (16.6), 13 | 59.4 (14.9), 13 |
| Geriatric Depression Scale (short form) | 5.4 (4.5), 13 | 4.8 (3.5), 13 | 4.8 (3.8), 13 | 4.3 (3.7), 13 |
| McGill Pain Questionnaire | 22.5 (14.5), 13 | 34.3 (13.2), 12 | 25.1 (15.1), 13 | 23.7 (17.9), 11 |
| Pain Self-Efficacy Questionnaire | 41.8 (15.0), 13 | 36.5 (13.9), 13 | 40.7 (17.1), 13 | 41.9 (13.8), 13 |
| Pain intensity (visual analogue scale) | 4.2 (2.8), 13 | 5.8 (2.2), 13 | 4.7 (2.5), 13 | 4.9 (2.2), 13 |
| Brief Illness Perception Questionnaire | 41.8 (14.8), 13 | 46.3 (12.4), 13 | 40.8 (13.4), 13 | 43.5 (9.8), 13 |

Additional Table 2: All outcome measures at follow-up (3 months). All presented as Mean (SD), N.

|  | **TENS + PE** | **TENS** | **Placebo TENS + PE** | **Placebo TENS** |
| --- | --- | --- | --- | --- |
| **Walking distances** | | |  |  |
| Initial Claudication Distance (ICD) (metres) | 173.2 (281.0), 10 | 143.9 (158.1), 13 | 261.8 (285.1), 12 | 230.3 (319.0), 10 |
| Absolute Claudication Distance (ACD) (metres) | 311.1 (313.7), 10 | 284.0 (242.7), 13 | 426.9 (312.6), 12 | 284.1 (299.9), 10 |
| **Physical activity measures** |  |  |  |  |
| Mean daily number of steps | 4719 (2737), 5 | 6356 (7343), 10 | 6753 (7424), 8 | 3853 (3184), 8 |
| Mean daily number of upright events | 42.2 (12.8), 5 | 38.5 (11.2), 10 | 36.8 (18.4), 8 | 30.0 (8.0), 8 |
| Mean daily number of walking events | 256.1 (90.7), 5 | 260.2 (132.8), 10 | 260.3 (141.1), 8 | 260.4 (173.1), 8 |
| Mean Event-Based Claudication Index | 6.2 (1.8), 5 | 6.6 (2.3), 10 | 7.7 (3.9), 8 | 8.7 (5.9), 8 |
| **Participant-reported measures** | | | |  |
| SF-36 Physical Component Score | 34.6 (5.5), 11 | 33.3 (8.7), 13 | 34.0 (6.8), 13 | 33.3 (8.5), 10 |
| SF-36 Mental Component Score | 26.6 (10.9), 11 | 23.3 (10.0), 13 | 22.5 (12.4), 13 | 21.4 (9.4), 10 |
| Intermittent Claudication Questionnaire | 56.6 (14.0), 11 | 57.3 (15.3), 13 | 60.4 (17.3), 13 | 58.0 (13.3), 10 |
| Geriatric Depression Scale (short form) | 6.3 (4.5), 11 | 5.4 (4.1), 13 | 4.5 (3.6), 13 | 4.5 (3.6), 10 |
| McGill Pain Questionnaire | 26.2 (13.6), 10 | 32.8 (13.2), 13 | 23.3 (14.0), 12 | 22.9 (18.5), 10 |
| Pain Self-Efficacy Questionnaire | 36.8 (12.6), 11 | 37.4 (15.7), 13 | 41.3 (15.4), 13 | 44.6 (12.5), 10 |
| Pain intensity (visual analogue scale) | 5.5 (3.3), 11 | 5.2 (2.4), 13 | 4.9 (3.1), 13 | 4.2 (2.0), 10 |
| Brief Illness Perception Questionnaire | 43.5 (13.0), 11 | 46.5 (10.8), 13 | 45.3 (11.7), 13 | 42.9 (8.1), 10 |

Additional Table 3: Change scores from baseline to end of intervention: Mean (95% CI)

|  | **TENS + PE** | **TENS** | **Placebo TENS + PE** | **Placebo TENS** |
| --- | --- | --- | --- | --- |
| **Walking distances** | | | | |
| Initial Claudication Distance (metres) | 52.2 (5.0, 99.4) | 37.8 (5.9, 69.8) | 107.6 (37.3, 178.0) | 23.2 (-117.0, 163.5) |
| Absolute Claudication Distance(metres) | -0.9 (-44.2, 42.4) | -16.8 (-62.1, 28.4) | 76.2 (17.0, 135.4) | -6.7 (-33.8, 20.5) |
| **Physical activity measures** | | | | |
| Mean daily number of steps | 442 (-430, 1314) | -1568 (-3766, 631) | 1076 (-758, 2910) | -237 (-874, 399) |
| Mean daily number of upright events | 1.7 (-2.3, 5.7) | -1.1 (-8.6, 6.3) | -3.9 (-13.5, 5.6) | -2.3 (-6.9, 2.4) |
| Mean daily number of walking events | 16.3 (-46.1, 78.6) | -27.7 (-88.4, 33.0) | -27.6 (-106.1, 50.8) | -22.1 (-65.3, 21.1) |
| Mean Event-Based Claudication Index | -0.2 (-1.1, 0.7) | -0.4 (-1.2, 0.3) | 0.9 (-1.0, 2.7) | -0.4 (-1.1, 0.3) |
| **Participant-reported measures** | | | | |
| SF-36 Physical Component Score | -4.5 (-8.4, -0.7) | 1.4 (-1.4, 4.3) | -5.2 (-8.5, -1.8) | 0.0 (-3.3, 3.3) |
| SF-36 Mental Component Score | -1.8 (-6.8, 3.2) | -2.8 (-7.6, 2.0) | -3.0 (-9.4, 3.4) | 0.5 (-5.9, 7.0) |
| Intermittent Claudication Questionnaire | 9.6 (3.5, 15.7) | 4.5 (-1.3, 10.2) | 8.5 (1.7, 15.3) | 5.6 (0.3, 10.8) |
| Geriatric Depression Scale (short-form) | -1.2 (-2.6, 0.2) | -1.5 (-3.3, 0.2) | -1.8 (-4.0, 0.3) | -0.8 (-3.5, 2.0) |
| McGill Pain Questionnaire | -6.5 (-12.3, -0.6) | 4.3 (-0.8, 9.5) | -5.9 (-9.9, -1.9) | 3.5 (-4.0, 11.1) |
| Pain Self-Efficacy Questionnaire | 6.9 (1.7, 12.1) | 1.0 (-4.2, 6.2) | 5.2 (-1.5, 11.8) | 0.8 (-2.5, 4.2) |
| Pain intensity (visual analogue scale) | -1.5 (-3.1, 0.0) | -0.7 (-2.2, 0.8) | -2.0 (-3.3, -0.7) | -1.0 (-2.4, 0.3) |
| Brief Illness Perception Questionnaire | -8.1 (-15.1, -1.0) | -1.7 (-6.8, 3.5) | -7.7 (-12.8, -2.5) | 0.6 (-5.0, 6.3) |

Additional Table 4: Change scores from baseline to 3-month follow-up: Mean (95% CI)

|  | **TENS + PE** | **TENS** | **Placebo TENS + PE** | **Placebo TENS** |
| --- | --- | --- | --- | --- |
| **Walking distances** | | | | |
| Initial Claudication Distance (metres) | 54.1 (-3.9, 112.1) | 55.2 (-11.4, 121.7) | 122.7 (26.6, 218.9) | 77.1 (-83.4, 237.5) |
| Absolute Claudication Distance (metres) | -15.1 (-64.1, 33.9) | 25.0 (-26.8, 76.7) | 89.2 (21.3, 157.1) | -17.6 (-41.0, 5.8) |
| **Physical activity measures** | | | | |
| Mean daily number of steps | -1637 (-4010, 736) | -1942 (-3239, -646) | 1615 (-2425, 5654) | -183 (-1048, 683) |
| Mean daily number of upright events | -3.4 (-13.0, 6.1) | -2.1 (-8.4, 4.1) | -4.7 (-9.2, -0.2) | 2.5 (-3.8, 8.9) |
| Mean daily number of walking events | -39.4 (-107.6, 28.8) | -53.5 (-99.6, -7.4) | -36.9 (-98.7, 25.0) | 0.1 (-75.8, 76.1) |
| Mean Event-Based Claudication Index | -0.6 (-1.0, -0.2) | -1.2 (-2.5, 0.1) | 0.0 (-1.0, 1.0) | -0.7 (-3.2, 1.7) |
| **Participant-reported measures** | | | | |
| SF-36 Physical Component Score | -3.3 (-7.2, 0.7) | -0.6 (-3.4, 2.2) | -3.7 (-5.5, -1.8) | 1.0 (-2.6, 4.6) |
| SF-36 Mental Component Score | -0.4 (-4.7, 4.0) | -0.1 (-4.4, 4.3) | -2.7 (-7.2, 1.7) | 0.1 (-7.8, 8.0) |
| Intermittent Claudication Questionnaire | 8.0 (3.2, 12.7) | 4.4 (-0.4, 9.2) | 9.3 (1.9, 16.8) | 2.3 (-3.9, 8.5) |
| Geriatric Depression Scale (short-form) | -1.0 (-2.3, 0.3) | -0.9 (-3.2, 1.3) | -2.2 (-4.8, 0.4) | 0.2 (-2.8, 3.2) |
| McGill Pain Questionnaire | -3.0 (-8.1, 2.1) | 2.5 (-1.3, 6.3) | -7.4 (-11.0, -3.8) | 2.6 (-2.4, 7.6) |
| Pain Self-Efficacy Questionnaire | 3.6 (-1.9, 9.1) | 1.9 (-3.7, 7.6) | 5.8 (-1.8, 13.4) | 0.4 (-5.5, 6.3) |
| Pain intensity (visual analogue scale) | 0.1 (-1.4, 1.6) | -1.2 (-2.7, 0.2) | -1.8 (-3.1, -0.4) | -1.4 (-3.4, 0.6) |
| Brief Illness Perception Questionnaire | -5.6 (-10.9, -0.3) | -1.5 (-6.2, 3.1) | -3.2 (-8.9, 2.4) | 1.0 (-5.3, 7.3) |
