# Supplementary material for: Pain management and patient education interventions to increase physical activity in people with intermittent claudication (PrEPAID): a feasibility randomised controlled trial in the UK
Source: BMJ Open. 2025 Jul 22;15(7):e105563. doi: 10.1136/bmjopen-2025-105563 (PMC12306470; doi:10.1136/bmjopen-2025-105563)
Supplement: online supplemental file 2 [file bmjopen-15-7-s002.docx]

**PrEPAID Education Follow Up Phone Calls**

| Participant ID: |  |
| --- | --- |
| Date of call: |  |
| Researcher conducting call: |  |

Although there are questions to answer on this proforma try to make the conversation interactive with open questions which allow you to answer the key questions as the conversation progresses.

Hello my name is ....................

I am ringing on behalf of the PrEPAID study. You may remember that after you attended the PrEPAID workshop we said that we would follow up with a call to see how you are getting on and offer any additional support.

**1. Did you try to increase or change your walking exercise as a result of the workshop?**

Yes – Move to Qu 2 No – move to Qu 4

**If the person HAS changed behaviour and is doing well, ask:**

**2. What did you change in relation to your walking exercise?**

Explore context, what they changed, how, what helped/hindered them, outcomes of change (feel better etc)

What changes?

**3a. Have you reviewed your goals?**

Yes – provide details:

No – provide details:

**3b. Do you think you will continue to increase your walking exercise?**

Yes – go to Qu 5 No

**If the person HAS NOT changed behaviour or is no longer trying to walk more, ask:**

**4. To help us understand why you did not change your behaviour; can you identify any reasons why?**

What has gone less well? What has got in the way?

What would have to happen for you to start walking more?

How do you think you could do that?

If the person appears to be ready for change you may consider going through an action plan with the person e.g., what could you do, how much, when could you do it, what might stop you, what might help you, how confident do you feel?

What support might you need to achieve this?

If they are not ready to change, do not try and force a change. Accept the person’s view point and remind them that you are on hand if at any time they wish to call for advice or support.

***PEDOMETER***

At the workshop we gave you a step counter to help monitor your walking exercise, ask:

**5. Have you used the step counter in the last 2 weeks?**

Yes No – move to Qu 11

**6. What sort of experience have you had with this?**

**How is it helping? /Why hasn’t it helped you?**

**7. Have you used the step counter:**

Still using

Initially for ____ days/weeks but not now

Not at all – move to Qu 11

**8. How many days a week did you wear the step counter *in the first week*?**

Everyday

>3 days a week

<3 days a week

Never

**9. How many days a week do you wear the step counter *now*?**

Everyday

>3 days a week

<3 days a week

Never

**10. Did it help you to walk more?**

Yes No

***EXERCISE LOG***

At the workshop we gave you an exercise diary to help monitor your walking exercise, ask:

**11. Have you used the exercise diary in the last 2 weeks?**

Yes No – move to Qu 17

**12. What sort of experience have you had with this?**

**How is it helping? /Why hasn’t it helped you?**

**13. Have you used the exercise diary:**

Still using

Initially for ____ days/weeks but not now

Not at all – move to Qu 17

**14. How many days a week did you use the exercise diary *in the first week*?**

Everyday

>3 days a week

<3 days a week

Never

**15. How many days a week do you use the exercise diary *now*?**

Everyday

>3 days a week

<3 days a week

Never

**16. Did it help you to walk more?**

Yes No

Finishing the call. Just one final question:

**17. If you had a friend who had intermittent claudication, would you recommend the workshop we ran to them? Why?**

Yes No

Finally, if you need any support please remember that you can call me or Lesley at any time.

Thank you
