# Supplementary material for: Pain management and patient education interventions to increase physical activity in people with intermittent claudication (PrEPAID): a feasibility randomised controlled trial in the UK
Source: BMJ Open. 2025 Jul 22;15(7):e105563. doi: 10.1136/bmjopen-2025-105563 (PMC12306470; doi:10.1136/bmjopen-2025-105563)
Supplement: online supplemental file 3 [file bmjopen-15-7-s003.docx]

**DATA COLLECTION SHEET FOR FOCUS GROUP VISIT**

***Information for focus group facilitators: This session should be facilitated by two persons. Prior to focus group session, all materials including audio tape should be ready and available. Make sure minimum number of sample (4 participants) is participating in each session. The focus group will be implemented according to the topic guide below.***

**FOCUS GROUP TOPIC GUIDE**

**INTRODUCTION**

Introduce focus group moderators, participants, and purpose of discussions:

“The aim of this discussion is to elicit your experiences of living with PAD and IC and using TENS for walking, and/or participating in the education session. “

“Before we start, we would like to let you know that our discussion is recorded as part of the research process – are you happy to go ahead? The focus group interview will later be transcribed for analysis and anonymised so that your personal details and everything you say is kept confidential and cannot be identified. We will talk about your experiences and views of taking part in this study and [IF RELEVANT] your experience using TENS and/or the structured education programme you received. If that sounds ok, is there anything you would like to say or ask before I start the recorder?”

*Allow as many participants as possible to talk on each statement/question before moving to the next*

**BRIEF HISTORY**

“Before we begin to talk about the study.

- Could you tell me a bit about your PAD and IC from when you first became aware of your condition?”
- “What treatments have you received, and what you tried to do yourself, in the past to help manage your condition? How successful have these strategies/treatments been?”

**RESEARCH EXPERIENCE**

“What were your thoughts when you heard about the research?”

*Confirm mode of recruitment –face-to-face approach in clinic.*

*Collect feedback on whichever of the following are relevant to the participants’ mode of recruitment:*

- *Invitation letter*
- *Participant information sheet*
- *Organisation (location/timing of assessments)*

*Optional Prompts:*

- “Could there have been any additional information that might have been helpful for you at that point?
- Why did you choose to take part in the study?
- How did you find the study assessments at the hospital?
- Discuss: research setting; travel and timing; relationship with research nurse and clinical investigators; assessments (treadmill test, blood sample questionnaires, fixing of active PAL and ABPI test).
- How did you find using the activPAL at home?”

**INTERVENTION EXPERIENCE: (TENS)**

*Confirm which group the participant was in and what their initial preference was.*

- “What were your thoughts when you were told what group you had been allocated to?
- What were your expectations of the TENS machine before you start using it?
- What are your thoughts about using the TENS device? *(Discuss: frequency, time, intensity, and type of TENS (perceptible/non-perceptible stimulation))*
- What are your thoughts about the usability of TENS?
  - What was good?
  - What could have been improved?

*(Prompts: Instructions/ Controls/ Electrodes/ Appearance)*

- What were the challenges for you to continue to use the TENS as advised?
- What helped and encouraged you to use the TENS?
- How did you find the use TENS log at home?
- What changes would you recommend made to the intervention involving TENS if we were to run the study again?”

**INTERVENTION EXPERIENCE: (Education)**

*Confirm which group the participant was in and what their initial preference was.*

- “What were your thoughts when you were told what group you had been allocated to?
- What were your expectations of the structured education programme before you began the programme?
- What are your thoughts about the design of the structured education programme your received? *(Prompts: components, personnel, frequency, time and type of exercise)*
- What are your thoughts about the setting of the education programme, and the subsequent walking/exercise goals you set out? Was it appropriate? How could it have been better?
- What were the challenges for you to complete the education session, and to meet the physical activity/exercise set goals?
- What helped and encouraged you to complete the sessions, and to commit to the physical activity/exercise set goal?
- How appropriate did you find the use of pedometer in the programme?
- What changes would you recommend we made to the structured education programme if were to run the study again?

**OUTCOMES AND PROCESSES (ILLNESS AND TREATMENT BELIEFS)**

- How has the intervention affected you? [EXPLORE POSITIVE AND NEGATIVE CHANGES]
- What physical changes, if any, have you noticed as a result of the use of TENS and/or participating in the structured education programme?
- Apart from physical changes, how do you feel in yourself since using the TENS and/or participating in the structured education programme? (emotion)
- How do you see your condition (IC) now compared to before the using the TENS and/or participating in the structured education programme?
- Do you see your diagnosis/condition differently than before?
- Has your understanding of your condition changed?
- Has it affected how you will manage your condition in the future?

**THERAPEUTIC RELATIONSHIP**

- Can you tell me about your relationship with the person/people who delivered the education programme/training to the use of TENS?
- How did this relationship compare to your relationships with other healthcare professionals you have seen about your condition?
- Is there any aspect of what they did which could have been better?
- Do you think that the right sort of person delivered the structured education/training to the use of TENS?

**ACCEPTABILITY**

- Overall, how acceptable do you think the structured education programme/Self-management of pain with the use of TENS and research study procedures were?
- Would you recommend this sort of intervention (Self-management of pain with the use of TENS and/or structured education programme) or research study to other people with PAD/IC?
- What reasons do you think other people might have for not wanting to take part in this type of intervention (Self-management of pain with the use of TENS and/or structured education programme) or research study?
- Do you think that self-management of pain with the use of TENS and/or structured education programme should be offered on the NHS for people with PAD/IC? If so, who would deliver it and what would it look like?
- If self-management of pain with the use of TENS and/or structured education programme was available, but you had to pay for it, how much would you be willing to pay?

**CLOSING**

I believe we have now covered all the necessary topics. Is there anything else you would like to discuss either about what has happened or this research? Or do you have any questions?

Thank you for again your help*.*
