# Supplementary material for: Pain management and patient education interventions to increase physical activity in people with intermittent claudication (PrEPAID): a feasibility randomised controlled trial in the UK
Source: BMJ Open. 2025 Jul 22;15(7):e105563. doi: 10.1136/bmjopen-2025-105563 (PMC12306470; doi:10.1136/bmjopen-2025-105563)
Supplement: Supplementary file 4 [file bmjopen-2025-105563.reviewer_comments.pdf]

PEER REVIEW HISTORY

BMJ Open publishes all reviews undertaken for accepted manuscripts. Reviewers are asked to complete a checklist review form and are provided with free text boxes to elaborate on their assessment. These free text comments are reproduced below.

ARTICLE DETAILS

Title (Provisional)

Pain management and patient education interventions to increase physical activity in people with intermittent claudication (PrEPAID): a feasibility randomised controlled trial in the United Kingdom

Authors

Seenan, Chris; Abaraogu, Ukachukwu; Dall, Philippa Margaret; Gilmour, Lesley; Tew, Garry; Stuart, Wesley; Elders, Andrew; Brittenden, Julie

---

VERSION 1 - REVIEW

**Reviewer**	**1**
**Name**	**Chengxin Weng**
**Affiliation**	**Sichuan University West China Hospital Department of Vascular Surgery**
**Date**	**04-Dec-2024**
**COI**	**None**

Comments to the Author:

The improvement of walking distance and quality of life among individuals with peripheral artery disease and intermittent claudication has always been the most concerned issue for both medical workers and patients. Patient-centered education and TENS were an interesting combination that could potentially benefit patients with PAD/IC.
As a pilot study for a future large-scale RCT, this trial indeed explored the feasibility of the research process and explored the acceptance of interventions. However, there were still some problems.
1. Though this study reported some results of participant interviews, quantitative or qualitative analysis of the data in process evaluation part was still lacking. From general description of the interview, readers did not have a good understanding of the factors that influence participant recruitment, retention rates, adherence and their weights. A primary analysis result as well as targeted improvement measures may be necessary.
2. Primary outcome measures and sample size also affected feasibility of the trial. The current sample size does not seem appropriate due to the large variation in ACD within groups. In addition, unbalanced baseline between the intervention groups and control groups (e.g., smoking) might have influenced the results. The dynamic randomized algorithm could be used in the future study.
3. Moreover, sample size calculation was based on effect size of standard deviation for ACD, the current sample size would clearly not be appropriate if primary outcome changes. The lack of analysis and comparison of differences between groups also made it difficult to justify the selection for the primary outcome and sample-size calculation. Therefore, preliminary hypothesis testing and statistical analysis of the study results are still recommended.

In addition, there are some minor issues
1. Page7 line151, Please describe the 'stable IC' in the article.
2. Page9 line195, Whether a structured questionnaire was used here to follow up the participant?
3. The Cohen's d_s was used in the tables, Please describe the 'Cohen's ds' in the method part.

Reviewer **2**

Name **Miss Maram Darwish**

Affiliation **Cardiff and Vale University Health Board, Health Education East Midlands**

Date **04-Dec-2024**

COI **None**

Comments to the Author:
I read with great attention and interest the paper entitled " Pain management and patient education interventions to increase physical activity in people with intermittent claudication (PrEPAID): a feasibility randomised controlled trial "
The Authors describe a very interesting feasibility study; in extending my best wishes to the authors for the continuation of their project, I have a few points to emphasise:

The abstract:
PAD/IC should be spelled out in its first appearance in the abstract for clarity.

The introduction:
The introduction is overly lengthy and could benefit from being more concise. Typically, the introduction is expected to be around 5 paragraphs. Many sections can be moved to the discussion for instance. Streamlining this section will help maintain reader engagement and improve overall readability.

Objectives:
The paragraph "To achieve these aims the objectives of the study included: a) To estimate rates of recruitment, 138 retention, intervention uptake and adherence, and outcome completion for a definitive RCT; b) To 139 monitor, record, manage and follow-up specified adverse events in all groups; c) To collect and 140 synthesise data, including walking outcomes, physical activity and patient reported outcomes, 141 PROMs, from which the sample size of a definitive RCT could be estimated; d) To explore 142 participants' experiences and perceptions of the interventions and trial procedures via embedded 143 process evaluation" needs to paraphrasing to improve readability. For instance, it doesn't read right " To achieve these aims the objectives of the study included "To estimate"…" Please revise throughout to enhance clarity and readability.

Methods:
• The methods are well planned and explained thoroughly. However, while the study protocol understandably notes "Planned surgical or endovascular intervention for PAD within the next 3 months" as an exclusion criterion to participation. The authors did not mention if any of the participants has had any vascular intervention in the index limb in the past.

Results:
• Part of the study period was during the COVID pandemic, there is no mention about the confounding effect this, or the COVID vaccination, might have had on the results due to restrictions on physical activity as per the governmental rules at that time.

• 50% of the treatment group were active (current smokers) while only 7% of the placebo group were active (current smokers). I believe that might have created some bias and should be acknowledged as

so.

• Process evaluation: The list of the themes identified from patients' interviews is quite word heavy. Could this be better displayed graphically as a figure or at least as a table?

Limitations:
• Study Limitations should be presented separately with a separate headline for clarity, and not imbedded in the discussion.

Additional tables:
Table 1, table 3, and table 4 provided contain tracked changes/comments. Please revise and upload clean versions.

VERSION 1 - AUTHOR RESPONSE

Reviewer: 1	
1. Though this study reported some results of participant interviews, quantitative or qualitative analysis of the data in process evaluation part was still lacking. From general description of the interview, readers did not have a good understanding of the factors that influence participant recruitment, retention rates, adherence and their weights. A primary analysis result as well as targeted improvement measures may be necessary.	Thank you for your insightful comment. We acknowledge that the qualitative data does not provide much further contextualisation of the recruitment and adherence data presented in the quantitative results.<br><br>We chose to focus on acceptability of the interventions and study design within the interview discussions (see the topic guide, which is now included in supplementary material) and thus the interviews did not elicit information regarding recruitment, retention and adherence. We have added more discussion of this within the limitations to indicate areas for development in future trials, which now reads [lines 419-426]:<br><br>"**however, several limitations related to the process evaluation should be acknowledged. The process evaluation did not comprehensively explore participants' experiences with recruitment and consent procedures. Although retention rates were high, the exclusion of 40% of screened individuals due to the ACD variability criterion suggests that future studies should further examine how recruitment strategies and eligibility criteria impact participation. Semi-structured interviews did not specifically include questions on the recruitment process, meaning valuable insights into participants' decision-making, motivations, and perceived barriers to participation may have been overlooked**."
2. Primary outcome measures and sample size also affected feasibility of the trial. The current sample size does not seem appropriate due to the large variation in ACD within groups. In addition, unbalanced baseline between the intervention groups and control	Thank you for your comment.<br><br>The purpose of this trial was to assess feasibility of a future definitive trial, and we do not report efficacy outcomes in this study. We appreciate this was not clear in the original manuscript and have clarified the

groups (e.g., smoking) might have influenced the results. The dynamic randomized algorithm could be used in the future study.	aims of the study (see also the response to the editor above).<br><br>We therefore did not need to complete a sample size calculation a priori. Although not needed, we did perform a sample size calculation before the study, to sense-check the planned recruitment approach and ensure the feasibility of delivering our pilot study within available resources. We have now clarified this in the text, which reads [lines 178-181]:<br><br>"**As this is an external pilot study an a priori sample size calculation was not required. However, we did use a sample size calculation based on previous pilot data from the two arms of the intervention, to ensure that our selected sample size was suitable, minimising the burden of conducting the study while collecting sufficient data for future sample size calculations. [26]** At an 80% power and a two-tailed 5% significance level, the sample size calculation indicated that 64 participants (16 per group) were needed to detect an effect size of 1.0 standard deviation for the primary outcome (ACD) in the Active TENS group compared to the Placebo TENS control, based on insights from previous pilot trials. [21,23]. To account for potential attrition, a recruitment target of 80 participants was set. [26]"<br><br>As we did not aim to assess efficacy, the sample size had minimal impact on reporting of the current study. However, you make a very pertinent point about how the results of this study will affect the sample size for a definitive trial of education and TENS in people with PAD. The identification of the variability in ACD within and between participants is a key finding that importantly informs design and development of future trials in this area.<br><br>We also recognise that baseline imbalances in some characteristics, such as smoking status, may have influenced results. While this is a common challenge in small feasibility studies, we will consider stratified or dynamic randomisation techniques in a future trial to improve baseline comparability. The insights gained from this feasibility study will directly inform refinements to study design, including eligibility criteria, primary outcome selection, and randomisation procedures for a future definitive trial.<br><br>We have changed the text to clarify these points.

	In the results we have added [line 258-260]<br>"**There were some other differences in lifestyle behaviours (e.g. smoking; 50% of participants in the TENS + PE group were current smokers compared to 7% in the Placebo TENS +PE group) and medical history, potentially due to small group sizes.**"<br><br>We have also added text to the limitations section [Lines 440-449]<br>"**Despite efforts to exclude participants with highly variable ACD, substantial intra-individual variability remained, this is a key finding which should inform future trial design. This highlights the need for a more behaviourally sensitive primary outcome measure may be appropriate, such as daily step count or illness beliefs, which may be more responsive to the intervention and better aligned with behaviour change mechanisms. There was substantial baseline imbalance in some outcomes, for example smoking status, which may have biased the results. Future studies could consider stratified or dynamic randomisation techniques to improve baseline comparability. These limitations underscore key areas for refinement in future trials. Enhancing recruitment process evaluation, adherence tracking and qualitative follow-up will strengthen the validity and generalisability of findings.**"
3. Moreover, sample size calculation was based on effect size of standard deviation for ACD, the current sample size would clearly not be appropriate if primary outcome changes. The lack of analysis and comparison of differences between groups also made it difficult to justify the selection for the primary outcome and sample-size calculation. Therefore, preliminary hypothesis testing and statistical analysis of the study results are still recommended.	The authors acknowledge that the current sample size was not designed to assess efficacy and would not be appropriate for a definitive trial, particularly if the primary outcome is changed. As clarified in the introduction and methods sections, this study was conducted as an external pilot trial with the primary aim of assessing feasibility rather than testing intervention effectiveness. If the primary outcome for a future definitive trial changes from ACD, as suggested in the discussion, we will conduct a new sample size calculation tailored to the revised measure. If the new primary outcome is one of the secondary outcomes assessed in this study, the sample size estimation will be based on the variability observed in our current dataset.<br><br>We have added text to the discussion on lines 410-416<br>"These data suggest a review of primary outcome selection might be warranted for a future RCT and a potential shift to a 'proximal indicator' of the outcome of the interventions. This would align with recent research examining the effects of behavioural interventions in PAD that have used the Illness Beliefs

	Questionnaire [43] or steps per day [44] as primary outcome in their RCTs. **If this takes place, future sample size calculation for a definitive trial would need to be based on data from the primary outcome selected."**
In addition, there are some minor issues<br>1. Page7 line151, Please describe the 'stable IC' in the article.<br>2. Page9 line195, Whether a structured questionnaire was used here to follow up the participant?<br>3. The Cohen's d_s was used in the tables, Please describe the 'Cohen's ds' in the method part.	1. We have now added an explanation of what we mean by 'stable IC': "stable IC **(self-reported walking distance not substantially changed within the past 3 months)**" [Line 117]<br><br>2. Please see additional edits in methods section "**This conversation was guided by the PrEPAID follow-up phone call proforma (see Supplementary File 1)."** [Line 164] and proforma included in supplementary material.<br><br>3. Now included in methods- Statistical Analysis section: "All outcomes are summarised descriptively at each timepoint, with the standard deviation being estimated alongside a confidence interval in line with current guidance on estimation of standard deviations from feasibility trials [35] **and Cohen's d effects size**." [Line 231]
Reviewer 2	
The abstract:<br>PAD/IC should be spelled out in its first appearance in the abstract for clarity.	Thank you, this has now been addressed.
The introduction:<br>The introduction is overly lengthy and could benefit from being more concise. Typically, the introduction is expected to be around 5 paragraphs. Many sections can be moved to the discussion for instance. Streamlining this section will help maintain reader engagement and improve overall readability.	We have reduced the length of the introduction section, which is now more concise and covers 4 paragraphs. We hope this has improved readability without too much loss of detail.
Objectives:<br>The paragraph "To achieve these aims the objectives of the study included: a) To estimate rates of recruitment, 138 retention, intervention uptake and adherence, and outcome completion for a definitive RCT; b) To 139 monitor, record, manage and follow-up specified adverse events in all groups; c) To collect and 140 synthesise data, including walking outcomes, physical	Thank you for this comment. We have condensed the text reporting our aims and objectives to a single paragraph, in the process we have removed this paragraph from the manuscript. The aims now read [Line 104]:<br><br>"The overall aims of the study were twofold: 1) to explore the feasibility **of conducting a definitive RCT of TENS with and without patient-centred education in people with PAD and IC** (i.e., recruitment and retention rates, **intervention uptake and** adherence, safety,

activity and patient reported outcomes, 141 PROMs, from which the sample size of a definitive RCT could be estimated; d) To explore 142 participants' experiences and perceptions of the interventions and trial procedures via embedded 143 process evaluation" needs to paraphrasing to improve readability. For instance, it doesn't read right " To achieve these aims the objectives of the study included "To estimate"…" Please revise throughout to enhance clarity and readability.	**outcome completion rates and collect outcome information to allow future** sample size **estimation**); and 2) to explore how acceptable **a) the interventions** (TENS and patient-centred education**) and b) the trial procedures are** to people with PAD and IC."
Methods:<br>• The methods are well planned and explained thoroughly. However, while the study protocol understandably notes "Planned surgical or endovascular intervention for PAD within the next 3 months" as an exclusion criterion to participation. The authors did not mention if any of the participants has had any vascular intervention in the index limb in the past.	Unfortunately, we did not collect this information in the study and so cannot report it in the results. We would consider collecting this in future studies.
Results:<br>• Part of the study period was during the COVID pandemic, there is no mention about the confounding effect this, or the COVID vaccination, might have had on the results due to restrictions on physical activity as per the governmental rules at that time.	All of the quantitative data collection had finished prior to COVID lockdown, the only data collection affected by COVID was the qualitative component. We have now clarified this in the methods [Line 207]:<br><br>"**All quantitative data collection and delivery of interventions were completed prior to the UK national lockdowns in March 2020. However,** it was not possible to arrange **the** planned focus group**s** to explore participant experiences of the interventions and study procedures. Individual, semi-structured interviews were conducted via phone as an amendment to the protocol."
• 50% of the treatment group were active (current smokers) while only 7% of the placebo group were active (current smokers). I believe that might have created some bias and should be acknowledged as so.	We acknowledge that baseline imbalances in some characteristics, such as smoking status, may have influenced results. This is a common challenge in small feasibility studies. We have added text to the results [Line 258] and in the limitations section [Line 444]:<br><br>"**There were some other differences in lifestyle behaviours (e.g. smoking; 50% of participants in the TENS + PE group were current smokers compared to**

	**7% in the Placebo TENS +PE group) and medical history, potentially due to small group sizes.**"<br><br>"**There was substantial baseline imbalance in some outcomes, for example smoking status, which may have biased the results. Future studies could consider stratified or dynamic randomisation techniques to improve baseline comparability.**"
• Process evaluation: The list of the themes identified from patients' interviews is quite word heavy. Could this be better displayed graphically as a figure or at least as a table?	Thank you for this comment. We have created an overview of the findings of the process evaluation in table 4. We have also summarised the domain descriptions for the theoretical framework of acceptability in this table to clarify the intended content. Additionally, we now report our intended use of the TFA as the framework in our framework analysis in the methods [Line 217]
Limitations:<br>• Study Limitations should be presented separately with a separate headline for clarity, and not imbedded in the discussion.	Thank you for your useful comment. We have now added and clearly identified a limitations section [Lines 417 to 449]
Additional tables:<br>Table 1, table 3, and table 4 provided contain tracked changes/comments. Please revise and upload clean versions.	Thank you for spotting this and apologies we miss this in proof reading. It is now fixed.
